# Surgery of the Œsophagus and Stomach

**Published:** 1895-08-31

**Authors:** 


### SURGERY OF THE ŒSOPHAGUS AND STOMACH.

**Foreign Bodies in the Œsophagus** may be removed through the natural channels or by external œsophagotomy. Egloff,[1] as the result of his observations of six cases of œsophagotomy in Krönlein's clinique, of which only one died, thinks that recourse should be had to this operation the moment other means fail to afford relief. When the foreign body is pointed or angular, attempts at extraction should be made with great care in order to avoid inflicting injuries on the œsophagus, which are much more dangerous than external œsophagotomy.

**Cicatricial Stricture of the Œsophagus** has been treated by retrograde dilatation in twenty-eight cases, and Woolsey[2] after studying these cases concludes: (1) That if it is possible to dilate the stricture sufficiently in one sitting, the wound of the stomach should be closed immediately. If gastrostomy instead of gastrotomy is done, as it has been in seventeen out of the twenty-eight cases, the operation may be and is done in either one or two sittings. (2) That the opening of the stomach is best done in one sitting. The size of the opening depends partly upon how the dilatation is to be done and the after treatment of the case. If either Loreta's or Abbe's methods are employed, the opening should be large enough to allow the easy introduction of one or two fingers besides dilating instruments. Leakage may be minimised by making the opening as high up on the anterior wall and as near the cardia as possible; a position which is also the best for dilating the œsophagus. (3) The opening into the stomach should be of sufficient size to allow of the use of the finger in examination and in guiding instruments to the cardiac orifice of the œsophagus.

Bernays and Carter[3] record a case in which both gastrostomy and œsophagostomy were performed before the stricture became permeable. The author advocates the use of a "rosary bougie" in these cases. Winslow[4] has treated two cases of cicatricial stenosis of the œsophagus by gastrostomy. In one case the stricture was satisfactorily treated by retrograde dilatation, and the wound in the stomach was closed. In the other case it was necessary to perform œsophagotomy as well before the stricture was overcome, but the patient subsequently died.

**Gastrostomy.**—Sindner[5] gives the results of his experience of Frank's method of performing this operation, which consists in opening the abdominal cavity by a small incision; in drawing through the opening as large a portion as possible of the stomach; in fixing the base of this to the peritoneal edges of the wound, and in passing the free outer portion under a bridge of undermined skin, and finally bringing it through a second wound made over the margin of the ribs, to the edges of which the gastric walls are fixed by a second row of sutures. This operation is, in the author's opinion, superior to that of Hahn, in which there is a risk of wounding the pleura. The food can be retained, and also the gastric juice, which in older methods is freely discharged from the fistula, and causes deep ulceration of the skin around the margins of the wound in the abdominal wall. If the operation is performed for malignant disease of the œsophagus there is a fair expectation of a prolongation of life for at least twelve months under relatively comfortable conditions.

**Operative Treatment of Gastric Ulcer.**—Küster[6] has had two cases of gastric ulcer which were successfully treated by incision of the stomach, thermic cauterization, and gastro-enterostomy. In one case the progress towards recovery was much disturbed by frequent distension of the stomach by abundant yellow fluid, for the removal of which it was found necessary to wash out the organ very frequently in the course of the first five days following that of the operation. He draws the following conclusions: (1) Hæmorrhage from a gastric ulcer may be arrested by a single application of the actual cautery; (2) when the gastric ulcer is situated near the pylorus, gastro-enterostomy is preferable to pyloro-plasty, as the latter will not prevent cicatricial contraction and stenosis; (3) a wide anastomotic opening between the stomach and intestine is in no sense a disadvantage, but on the contrary will ensure the patient against the risks of undue contraction later. Rochemont[7] relates the following interesting case bearing on the etiology of this disease. A woman, aged thirty-eight, was admitted with gastric carcinoma. An examination of the gastric contents showed the presence of large quantities of lactic, but only traces of hydrochloric, acid. Gastro-enterostomy was performed, but the patient subsequently died with symptoms of perforative peritonitis. There was then found a large carcinoma on the lesser curvature of the stomach, and near the pylorus there was a funnel-shaped ulcer about the size of a three-mark piece. This latter ulcer was absolutely independent of the carcinoma, as was proved microscopically and otherwise, and there was sufficient evidence, in the author's opinion, to show that the ulcer appeared later than the carcinoma. Thus, hyper-acidity and increased digestive powers are not essential to the formation of a gastric ulcer; some other cause must be looked for in this case. A rather large thrombosed vessel was here found near the top of the ulcer. This had produced necrobiosis. It could not be ascertained whether this thrombosis had any relation to the carcinoma, that is, whether embolism had occurred from a thrombosed vessel in the region of the carcinoma. The aorta was atheromatous, and the heart muscle showed fatty degeneration. Jowers,[8] Lundie,[9] Kirkpatrick,[10] and Dunn[11] report successful cases of operation for perforating gastric ulcer, and the last also reports[12] an unsuccessful case of the same operation.

**Non-malignant Stricture of the Pylorus** has been treated by Ogston[13] by means of spheres of gutta-percha which the patient swallows, and which by their increasing size gradually bring about a dilatation of the pylorus. This operation is thought by the author to be safer than Loreta's or Mikulicz's operations. Gutta-percha spheres are easily made by rolling them in the hand into globules, and being of almost the specific gravity of the contents of the stomach have no tendency to settle in the lowest part of the stomach, or to float in the uppermost strata of its contents. The spheres are arranged into sizes by passing them through a gauge for urethral or œsophageal bougies. The gauges chosen were those on the French scale, but so sensitive is the pylorus that it was afterwards found convenient to mark intermediate sizes between two numbers; thus between, say, Nos. 20 and 21 would come 20 + and 21 —, the former being one that passed with difficulty through No. 20 aperture, the latter passing with ease through No. 21 aperture of the gauge plate. It was at first thought that the spheres would be most efficacious if given by the mouth at night; but they were found to cause sleeplessness from restlessness of the lower extremities ("fidgets") and bad nightmares, so that they were given just after breakfast, and the patient allowed to go about his usual avocations. The only precaution taken was to forbid very hot food and liquids, which might have softened the guttapercha so much as to destroy the rigidity of the sphere. They are found in the fæces, having passed through the alimentary tract without alteration. It is not difficult to form an approximate idea of the size of the pylorus in cases of stricture. If ordinary minced meat can be taken without discomfort, the stricture is certainly greater in size than a No. 10 or No. 20 urethral bougie of the French scale. If the orifice is less in size than this, only liquid and pulpy foods, containing no solid particles larger than boiled rice, can be taken without distress. These data are of assistance in commencing the treatment, which is best done by administering, immediately after breakfast, a sphere which is considerably less in size than the estimated size of the stricture, the patient swallowing it like a pill. If it causes no feeling of uneasiness in the pyloric region, a larger size should be given next morning, and so on. When the effective size has been reached it will be known by its causing within twelve hours uneasiness or soreness in the pyloric region, and perhaps characteristic cramp-like pains in the stomach.

After the size of the stricture has been determined, the treatment consists in administering successively larger spheres at intervals of about five days. The author has tried this treatment in four cases, but only one case was suitable for the treatment, which must be begun early. In this case 810 days were required to dilate the stricture from sixteen to forty millimetres of circumference, or from five up to fourteen millimetres diameter. The dilatation has not been pushed beyond 40 French bougie scale, as, with care, this is sufficient for the wants of the patient, who then suffers only when he eats unminced flesh. Morison[14] reports a successful case of pyloro-plasty after the Heinicke-Mikulicz plan in a woman, aged 48, who had been suffering for five years from vomiting and gastric pain.

**Perigastric Adhesions.**—Terrier[15] and Championnière[16] have both successfully divided adhesions between the stomach, liver, and omentum, which had given rise to recurrent symptoms of dyspepsia, vomiting, and great pain. Diagnosis is sometimes effected in these cases by the fact that the seizures occur about one hour after meals, and reach a climax two hours later. The only morbid antecedent that could in Championnière's case possibly account for the presence of the bands was an almost forgotten abscess of the liver, which had emptied itself into the gut.

**Carcinoma of the Stomach.**—Klemm[17] summarizes his opinion on the subject as follows: (1) The treatment of these tumours differs in no respect from that of cancer elsewhere. It is purely surgical. (2) Operation is to be recommended when possible, before the tumour is palpable. (3) Examination should only be made under deep narcosis, and an exploratory incision made if required. (4) Those cases only should be recommended for resection of the pylorus where the tumour is freely movable, and there is no metastatic involvement. (5) If these conditions are not present, the formation of a fistula between the stomach and jejunum is indicated. (6) This operation should not be delayed until the patient is nearly dead of hunger, and the knife then used as a last resource, for the mortality is then much greater. Langenbuch[18] has subjected three patients to total extirpation of the stomach for extensive carcinoma. Of these one only, a woman of fifty-eight, survived. In this case the portion removed represented seven-eighths of the stomach. The pylorus was stitched to the remains of the cardiac orifice, making a cavity about the size of a hen's egg. Fearing lest the sutures might give way, the new stomach was fixed in the abdominal wound by passing behind it a piece of iodoform gauze; the rest of the abdominal wound was closed. The operation lasted one and a half hour. Milk was given in the evening, and meat on the third day. Convalescence was rapid, and cure was accomplished in three weeks. Levy[19] describes an operation which he has devised for removing the œsophageal extremity of the stomach in cases of malignant disease. So far, the operation has been performed on cadavers and dogs only. The brief outlines of the operation are: (1) Incision through the abdominal wall, beginning in the middle line at the ensiform cartilage and extending to a finger's breadth below the navel, which is passed on its left side, and from this extremity of the wound a second incision is carried at a right angle through the right rectus muscle; (2) isolation of the cardia by cutting through the adjacent portion of the lesser omentum and applying double ligatures to the arteria and vena coronaria ventriculi sinistra; (3) isolation of the lower part of the œsophagus after dividing the serous covering around the cardia with scissors; (4) excision of the cardia and union of the cut surfaces. The œsophagus is divided, a portion at a time, and united to the stomach wound as it is cut. Without this precaution the divided end of the œsophagus disappears in the foramen œsophageum, and suture is then scarcely possible. Rosenheim[20] confirms the conclusions as to the presence of lactic acid not being a pathognomonic sign of cancer. He found it only in 55 per cent. of all cases. Lactic acid, therefore, is an inconstant phenomenon, this acid existing for a time, after which it subsides, to reappear later on. Lactic acid fermentation disappears more particularly as soon as the motor functions of the stomach are markedly improved, which is the case especially after performing gastro-enterostomy.

**Gastropexy.**—The operation devised by Duret for gastroptosis does not, in Le Dentu's[21] opinion, always do away with the symptoms in the immediate neighbourhood of, or at a distance from, this organ, depending upon its mobility or ptosis. Again, gastroptosis seldom exists alone, but frequently coincides with enteroptosis, gastrectasis, &c. In such cases gastropexy is obviously inadequate for effecting a cure. There are two other objections to this operation; in the first place, by fixing the pyolric end in the epigastric region, the viscus is forced to bend, seeing that the middle portion is not on the same level as the two extremities; secondly, the formation of adhesions between the stomach and abdominal wall must present certain inconveniences. For gastropexy, therefore, to offer any chance of success, it is necessary that the stomach should be merely displaced. It will always be called for occasionally, seeing that it is the last resource in a whole class of dyspepsias in which medical treatment is of no avail.

**Gastro-enterostomy** is now performed for non-cancerous affections, as well as for malignant diseases of the pylorus. Doyon[22] has operated on twenty-five cases. Eleven of these were for pyloric or duodenal stenosis, and of the remaining fourteen six, who had no appreciable ulcer, were completely cured of the dyspepsia. Kischkine[23] also reports a case of gastric dilatation cured by gastro-enterostomy.

**Gastro-anastomosis** for hour-glass contraction of the stomach caused by the scar of a gastric ulcer has been performed by Wölfler.[24] He opened the organ to the right and left of and below the scarred and contracted portion, and stitched the edges of the two orifices together, so as to establish a free communication between the dilated cardiac and the dilated pyloric portions of the stomach. Gastro-enterostomy would probably have failed on account of the atrophy and impaired function of the coats of the pyloric portion of the stomach.

[1] Med. Week, June 7, 1895, and Beitr. 7 Klin. Chir., XII., 1. [2] Annals of Surgery, March, 1895, p. 253, [3] New York Med. Journ., March 23, 1895, p. 353. [4] Annals of Surg., May, 1895. [5] Berl. Klin. Woch., No. 8, 1895, and Brit. Med. Jour., April 13, 1895. [6] Amer. Journ. of the Med. Sci., Feb. 1895, and New York Med. Rec., May 4, 1895. [7] Münch Med. Woch, Dec. 11, 1894, and Brit. Med. Journ., Jan. 26, 1895. [8] Lancet, March 2, 1895. [9] Brit. Med. Journ., Jan. 26, 1895. [10] Montreal Med. Journ., March, 1895, and Therapeutic Gazette, April 15, 1895. [11] Brit. Med. Journ., May 18, 1895, p. 1,093. [12] Ibidem. [13] Lancet, March 23, 1895, p. 739. [14] Lancet, Feb. 16, 1895. [15] New York Med. Rec., Mar. 23, 1895. [16] Ibidem. [17] St. Peter Med. Woch, 1894, No. 49, and Amer. Journ. Med. Sci., April, 1895. [18] La Trib. Med., Jan. 16, 1895, and Therap. Gazette, March 16, 1895. [19] Amer. Journ. Med. Sci., Feb., 1895. [20] Med. Week, Feb. 15, 1895. [21] Ibidem, March 22, 1895. [22] Therap. Gazette, Feb. 15, 1895. [23] Ibidem. [24] New York. Med. Journ., Feb. 23, 1895.

